# Plant Organelle Genome Replication

**DOI:** 10.3390/plants8100358

**Published:** 2019-09-21

**Authors:** Stewart A. Morley, Niaz Ahmad, Brent L. Nielsen

**Affiliations:** 1 Department of Microbiology & Molecular Biology, Brigham Young University, Provo, UT 84602, USA; stewart.morley@usda.gov; 2 Agricultural Biotechnology Division, National Institute for Biotechnology & Genetic Engineering, Jhang Road, Faisalabad, Punjab 44000, Pakistan; niazbloch@yahoo.com

**Keywords:** DNA replication, recombination-dependent replication (RDR), plant mitochondrial DNA, chloroplast DNA, DNA repair

## Abstract

Mitochondria and chloroplasts perform essential functions in respiration, ATP production, and photosynthesis, and both organelles contain genomes that encode only some of the proteins that are required for these functions. The proteins and mechanisms for organelle DNA replication are very similar to bacterial or phage systems. The minimal replisome may consist of DNA polymerase, a primase/helicase, and a single-stranded DNA binding protein (SSB), similar to that found in bacteriophage T7. In *Arabidopsis*, there are two genes for organellar DNA polymerases and multiple potential genes for SSB, but there is only one known primase/helicase protein to date. Genome copy number varies widely between type and age of plant tissues. Replication mechanisms are only poorly understood at present, and may involve multiple processes, including recombination-dependent replication (RDR) in plant mitochondria and perhaps also in chloroplasts. There are still important questions remaining as to how the genomes are maintained in new organelles, and how genome copy number is determined. This review summarizes our current understanding of these processes.

## 1. Introduction 

### 1.1. Discovery of Mitochondria and Chloroplasts

In 1665 Robert Hooke became the first person to observe cells with a simple microscope [1]. Almost one hundred and fifty years later in 1804 Franz Bauer described the discovery of the first observed organelle, the nucleus [2]. In 1890 Richard Altmann described what he called “bio-blasts,” or what we now call mitochondria [3]. Around the same time in 1883, A. F. W. Schimper described “chloroplastids”, what we now know as chloroplasts [4]. Both of these organelles house important biochemical reactions that are essential for cell survival; mitochondria generate ATP, and chloroplasts are the site of photosynthesis, and both house other important functions [3,5].

### 1.2. Evolutionary Origins of Each Organelle

Both mitochondria and chloroplasts are believed to have originated through endosymbiosis. Free-living aerobic α-proteobacterium-like cells taken up by a nucleus-containing (but amitochondriate) host cell gradually developed into mitochondria. Chloroplasts are also thought to have developed by a similar process, in which a eukaryotic cell (containing the mitochondria) engulfed a photosynthesizing prokaryotic cell, which eventually evolved into present-day chloroplasts. Over the course of evolution, both of these incoming cells entered into an endosymbiotic relationship with the host, synchronizing their own division with that of the host cell, transferring their genetic material to the host nucleus, and becoming permanent cellular structures of exogenous origin [5,6,7]. Despite the extensive knowledge of organelle genome structure, evolution and content that has been reported, there are still important questions regarding how these genomes are replicated, and the proteins and control mechanisms involved.

## 2. Organelle Genomes and Structure

### 2.1. Genome Size

**Figure 1:**
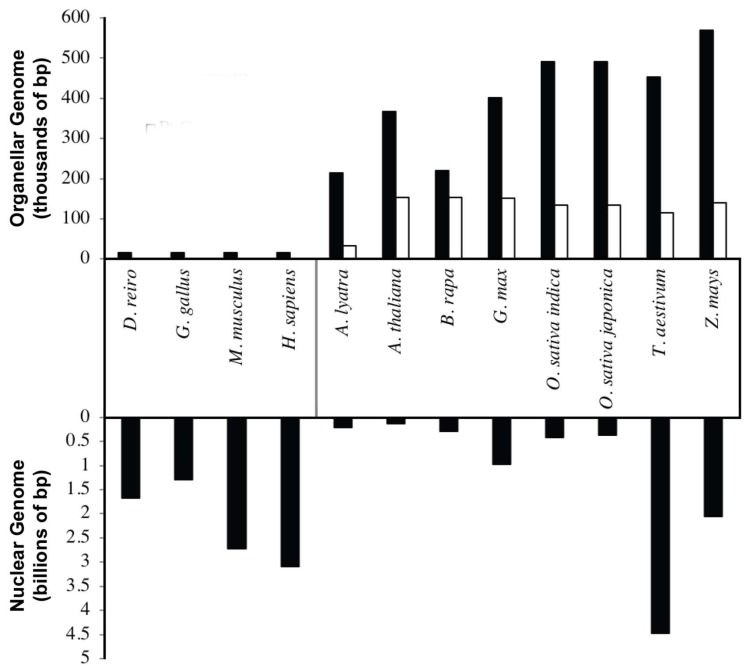
Nuclear and organelle genome sizes among different organisms. Mitochondrial genomes (dark bars in upper panel) among animals are compact and remarkably similar in size: ~16.5 kb. Plants, however, have mitochondrial genomes that dwarf those found in animals and vary in size from species to species. Chloroplast genomes vary less in size (open bars in upper panel) from organism to organism but still are relatively large compared to animal mitochondrial genomes. Organelle genome sizes do not correlate with nuclear genome size (bottom panel).

Endosymbiosis is accompanied with massive gene transfer to the nucleus of the host cell, resulting in considerable size reduction of the genome of the incoming cells. This is observed in the considerable size reduction in the mitochondrial and chloroplast genomes and the presence of mitochondrial and chloroplast DNA (cpDNA) sequences in the nuclear genomes of many plant species [5,8,9]. Mitochondrial genomes in plants have evolved very differently as compared to animal mitochondrial genomes (Figure 1). Most animal mitochondrial genomes are roughly 16 kb in size [10], and the number of genome copies per mitochondrion varies from study to study. Older estimates place as many as 10 copies per organelle [11], whereas more recent data suggests it may be as low as one [12]. Regardless of the actual number, mitochondrial genome copy number is thought to be tightly regulated in animal cells [13]. In contrast, plant mitochondrial genomes are much larger, and have tremendous size variations (187–2400 kb [14]) among different species. In addition, significant diversity in the number of copies of mitochondrial DNA (mtDNA) per organelle have been reported in different species, tissues and cell types [15,16]. The reasons for these copy number differences are unclear. 

Compared to mitochondrial genomes, chloroplast genomes are fairly uniform, ranging between 120 and 160 kb in size, with some exceptions being as large as 2000 kb [17,18]. Higher plant chloroplast genomes possess large inverted repeats varying between 22–24 kb, accounting for ~16% of the chloroplast genome [19]. Surprisingly, removal of these repeats is associated with a higher frequency of recombination events and fewer nucleotide substitution events [20,21]. It is thought that these large inverted repeats in higher plants are involved in maintaining fidelity and correction of mutations or reducing errors in the cpDNA [21,22].

This uniformity suggests that genome reduction in chloroplasts might have taken place in a relatively short period of time soon after endosymbiosis [22]. Cytological observations indicate that cpDNA copy numbers seem to be related to plastid size, plastid type, developmental stage of the plant, and tissue type, and cpDNA copy number estimates per chloroplast range from several hundred to nearly two thousand per organelle [23,24]. It has been observed that the amount of cpDNA increases during the conversion of proplastids to mature chloroplasts (development), but it decreases during senescence (aging) [25,26,27,28]. This implies that reduction in cpDNA level can be tolerated to some extent and still maintain organelle functionality. 

### 2.2. Genome Structure and Content

In both mitochondria and chloroplasts the DNA is associated with positively charged proteins in nucleoids [12]. Animal mtDNA consists of a singular circular molecule [29] and is very gene dense, with about 97% of the DNA coding for functional genes [30,31]. In most animals, the small non-coding region has important sequence elements for regulation of DNA replication and gene transcription [32]. One significant exception to this generalization can be seen in non-bilaterian animals, which possess large segments of non-coding DNA and varying levels of linear and circular DNA molecules [31].

For the most part, animal mitochondrial genomes encode the same 37 genes: two for rRNAs, 13 for proteins and 22 for tRNAs [10]. All 37 of these genes possess homologs in plants, fungi, and protists. To date, mtDNA gene content among animals only varies in nematodes [33], a bivalve [34], and cnidarians [35]. In these exceptions, there have been losses and gains of different mitochondrial genes, mostly tRNA genes.

Although plant mitochondrial genomes are mapped as circular molecules (master circles), circular molecules equal to a genome equivalent have only been observed in cultured liverwort cells [36]. Typically, plant mtDNA is observed primarily as large subgenomic linear molecules when observed by electron microscopy or pulsed field gel electrophoresis (PFGE). PFGE utilizes alternating direction of the current to allow separation of large DNA molecules or complex structures such as branched, lariat, rosette or catenated molecules, which are found in varying abundance in plant mtDNA preparations [37,38,39]. With PFGE, a large portion of the plant mtDNA remains trapped in highly complex arrangements near the wells [40]. Viewed by electron microscopy, these complex arrangements form DNA ‘rosettes’ and branched molecules, suggesting high levels of recombination. Other high molecular weight plant mtDNA simply does not enter the gel at all and has been theorized to be relaxed circle DNA, complex replication intermediates, or DNA bound to a matrix of other materials.

In contrast to animals, plant mtDNA contains many more genes and large portions of non-coding or undefined DNA [41]. A typical plant mitochondrial genome encodes anywhere between 50 and 100 genes [42]. The large genome size is at least partially due to the presence of non-coding DNA sequences, which consist of introns, repeats, and duplications of regions of the genome [41,43]. The known genes encode rRNA and tRNA genes as well as subunits for oxidative phosphorylation chain complexes [44]. The presence of these large non-coding DNA may have a role in lowering the mutation rate [45], as observed in *Arabidopsis thaliana* ecotypes Col-0 and C24. These two ecotypes have genetically identical mitochondrial genomes, but arrange their genes in different orders [46,47].

Chloroplast genomes exist primarily as homogeneous closed circle DNA molecules [48,49]. A small portion of these molecules also exist as circular dimers [19]. One exception to these observations can be seen among two species of brown algae [50]. In general, genes are conserved in most chloroplast genomes. For the most part, these consist of rRNA, tRNA, and genes involved in photosynthesis [17,18]. Loss of genes seems to be the only difference in gene content when comparing genomes. In these cases, essential genes have been lost from the chloroplast genome and transferred to the nucleus. Considering the possibility of multiple independent endosymbiotic events, it is interesting to observe the relatively conserved number and type of genes found in chloroplast genomes. 

## 3. Organelle DNA Replication

### 3.1. Models for Organelle DNA Replication

#### 3.1.1. Animal Mitochondria

**Figure 2:**
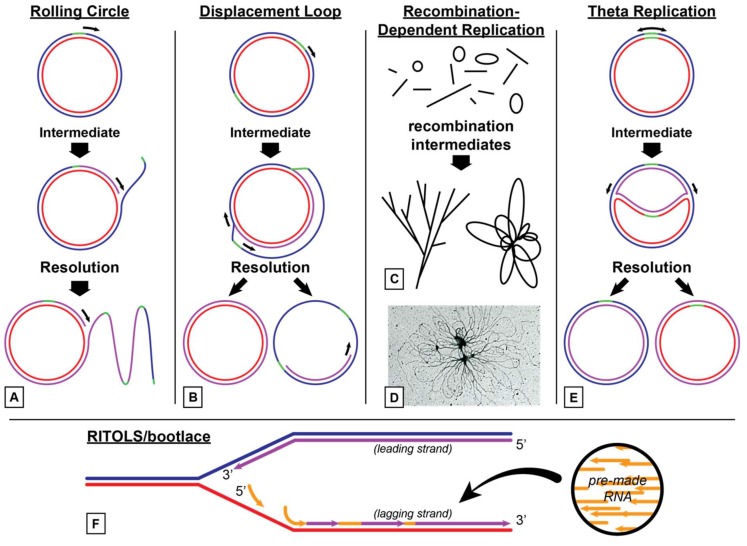
Proposed DNA replication mechanisms for mitochondrial DNA. (**A**) Rolling circle replication involves unidirectional replication after nicking of one DNA strand. DNA replication continues along the circular molecule displacing the nicked strand. Upon reaching the initial start site, the displaced strand may be nicked and ligated to form a new single stranded circular molecule or synthesis may continue, creating a linear concatemeric molecule which is later converted into multiple single stranded circular copies of the parent molecule. (**B**) Displacement loop (D-loop) replication proceeds unidirectionally by synthesis of an RNA primer that displaces one of the DNA strands. Upon synthesizing a certain portion of the genome (commonly 2/3) a second origin site is exposed as a single strand, which triggers DNA synthesis in the opposite direction. By the time the first double stranded DNA molecule is finished, synthesis on the parent strand is still ongoing. Once replication reaches the initial start site, the parent strand is displaced as a single stranded circular DNA molecule. The single stranded circular molecules formed by rolling circle and displacement loop replication are later turned into double stranded copies by DNA replication machinery. (**C**) Recombination-dependent replication (RDR) involves the use of many linear and circular pieces of DNA that share homology. These pieces recombine to form branched linear and “rosette” like intermediates that are copied and replicated by DNA machinery. (**D**) Electron micrograph image of DNA forming a “rosette” that is likely the result of recombination. (**E**) Theta replication is so named because of the intermediate it forms as a result of bi-directional DNA replication. Replication initiates bi-directionally at an origin of replication, forming two replication forks. When these replication forks meet, the two double stranded circular molecules are separated. (**F**) The RITOLS (Ribonucleotide Incorporation ThroughOut the Lagging Strand)/bootlace strategy of replication involves the lagging strand of a replication fork. While the leading strand replicates normally, free pre-synthesized RNA molecules in the mitochondria (indicated by the arrow) hybridize to the lagging strand of the mtDNA starting from the 3’ end of the RNA and proceeding in the 5’ direction. Gaps are filled in and the primers are removed by the DNA replication machinery.

Several modes of DNA replication in animals have been proposed (Figure 2). These include rolling circle, theta replication, strand-displacement, and RITOLS (Ribonucleotide Incorporation ThroughOut the Lagging Strand)/bootlace [51]. Rolling circle replication assures efficient reproduction of genomes exploiting a bacteriophage-like mechanism. Theta replication is the predominant replication mode among invertebrates, although nematodes have been observed to employ rolling circle DNA replication [52].

In vertebrates, two methods of mtDNA replication are currently accepted (Figure 2). The first is strand-displacement, or D-loop replication [53]. RITOLS/bootlace replication is a variation of D-loop replication. RITOLS was coined after scientists observed replication intermediates that were resistant to DNA endonucleases but sensitive to RNaseH [54].

Animals utilize a simple minimal DNA replisome for the replication of mtDNA. This replisome is made up of TWINKLE DNA helicase and DNA polymerase Polγ [55]. These two enzymes are somewhat processive and can synthesize molecules about 2 kb in length. The addition of single stranded binding protein (SSB) to TWINKLE and Polγ increases the processivity of this replisome to generate the genome sized molecules of 16 kb.

#### 3.1.2. Plant Mitochondria

Plants most likely employ multiple mechanisms for replication of the mtDNA due to the complex structure of the mitochondrial genome. The structure of plant mtDNA makes strand displacement (D-loop) replication implausible, although there is one report of this mechanism observed in petunia flowers [56]. Rolling circle replication has also been observed in *Chenopodium album,* suggesting it could be a common replication mode in other plant species as well [57,58]. However, it is not possible to predict the exact mechanism for plant mtDNA replication, due to the large amount of non-coding DNA and the complex DNA structures observed, as mentioned above. Many scientists have proposed, based on the available information, that the main methods plants use for mtDNA replication include recombination-dependent replication (RDR) and recombination independent rolling circle replication. The mitochondrial resolvase Cce1 has been shown to play a role in mtDNA segregation [59].

#### 3.1.3. Plant Chloroplasts

Replication of cpDNA is better understood than plant mitochondrial DNA replication. Chloroplasts utilize a double displacement loop strategy to initiate DNA replication [60]. The two displacement loops begin on opposite strands and begin replicating unidirectionally towards each other until they join to create a bidirectional replication bubble [61,62]. At this point, the displacement loops fuse, forming a Cairns or theta structure and DNA replication continues bidirectionally until two daughter molecules are created. Rolling circle and recombination-dependent replication have also been proposed for cpDNA [24,61,62]. MOC1 has been identified as a Holliday junction (recombination intermediate) resolvase that mediates chloroplast nucleoid segregation [63].

Some exceptions to the double D-loop replication model have been proposed. For example, *Chlamydomonas* and *Oenothera* possess two displacement loops, but discontinuous DNA replication begins shortly after initiation rather than after the fusion of the two D-loops [64,65]. *Euglena* possesses only one origin of replication site and appears to replicate bidirectionally from this site rather than forming two displacement loops [66].

### 3.2. Similarity to T7 Bacteriophage

**Figure 3:**
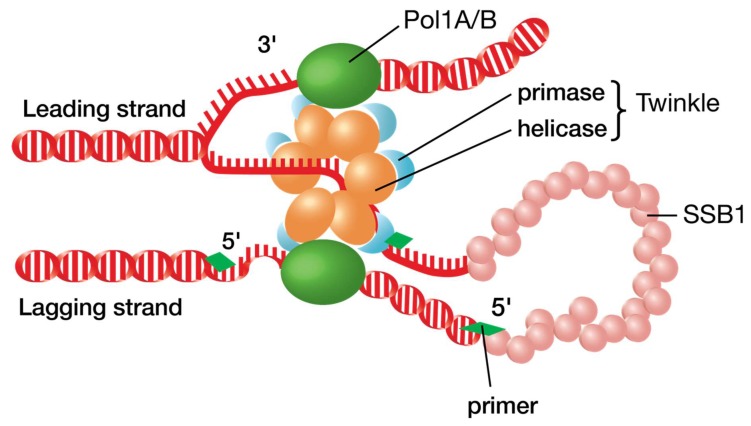
Theoretical model of the plant organellar DNA replisome. Four proteins are most likely involved in the minimal plant organellar DNA replisome, including Pol1A or Pol1B DNA polymerase, Twinkle DNA helicase/primase, and SSB1 single stranded binding protein. This model is similar to the replisome used by T7 phage which includes the proteins gp5 (DNA polymerase), gp4 (helicase/primase), and gp2.5 (single stranded binding protein). Adapted from Wikipedia file: phage T7 replication machinery.png, created 1 February 2015; https://creativecommons.org/licenses/by-sa/4.0/.

Plant organelles likely mimic the minimal DNA replisome of T7 phage (Figure 3). The T7 DNA replisome consists of proteins gp5 (T7 DNA polymerase), gp4 (DNA helicase/primase) and gp2.5 (DNA single stranded binding protein). *E. coli* thioredoxin also binds to gp5 to increase the processivity of the enzyme [67]. Animal mitochondria use a similar system consisting of DNA Polγ, Twinkle, and SSB1 protein [55]. Since plant organelles possess the same proteins, one could logically assume that the same replisome is tasked with maintaining and replicating DNA in chloroplasts and plant mitochondria. However, while Twinkle knockouts in animals are lethal, Twinkle knockouts in plants lead to no distinguishable phenotype. Genome copy numbers in organelles also remain unchanged (S.A.M. and B.L.N., unpublished data). This contradicts the idea that, like with phage T7 and in animal mitochondria, a Twinkle-Pol1A/B replisome is the main driver of DNA synthesis in plant organelles. This also highlights the likelihood of plants utilizing multiple methods to replicate the organellar genomes rather than depending on a single mechanism.

## 4. Proteins Involved in Plant Mitochondrial and Chloroplast DNA Replication

### 4.1. Organelle DNA Replication Proteins 

The genomes in both mitochondria and chloroplasts are complexed with positively charged proteins in nucleoids [12,68], and this is the form of the DNA that is replicated in the organelles. Key functions required for DNA replication include polymerization, DNA unwinding, priming, strand separation, recombination, and ligation. These functions are carried out by nuclear encoded proteins that target to either the mitochondria, chloroplasts, or both. For the sake of simplicity, we will only discuss those replication proteins described in *Arabidopsis,* as their homologs exist in all vascular plants (Table 1). An interesting point to mention is that DNA replication proteins in plant organelles have different phylogenetic sources. For example, the DNA polymerases are bacterial in origin, while Twinkle helicase-primase and RNA polymerases are phage-like. 

### 4.2. DNA Polymerases

To date, two organellar DNA polymerases, Pol1A and Pol1B, resembling bacterial DNA Pol1, have been discovered in both mitochondria and chloroplasts. Although Pol1A and Pol1B are similar to each other, notable differences between the two have been observed. Pol1B knockout plants were shown to have fewer genome copy numbers per organelle and grew slowly [90], whereas the ΔPol1B mutant showed increased sensitivity to double stranded DNA breaks, suggesting a predominant role of PolB in cpDNA damage repair [91]. Recent studies show that Pol1A replicates DNA with high fidelity and has an increased ability to displace DNA when replicating over short single stranded gaps of DNA [92,93]. Efforts to create a double mutant for both DNA polymerases has not been successful yet, suggesting the essentiality of these polymerases for plant survival. However, heterozygous plants containing a single copy of either Pol1A or Pol1B were able to grow to maturity. 

Both Pol1A and Pol1B have been shown to replicate an entire genome equivalent in mitochondria and chloroplasts with a greater efficiency than the microbial DNA Pol1 [91,94,95]. Interestingly, recombinant versions of *E. coli* DNA Pol1 were able to bind with thioredoxin, resulting in a dramatic increase in processivity [96]. It is quite possible that plant organelle DNA polymerases may also bind thioredoxin to achieve high processivity; however, this has yet to be shown experimentally.

Both Pol1A and Pol1B are able to bypass DNA lesions and continue replicating DNA [97,98]. These types of DNA polymerases do not replicate DNA with a high degree of fidelity as observed for human POLQ and yeast DNA Polymerases [97,98]. One possible explanation could be the presence of 3’-5’ proofreading exonuclease domains, which are absent in typical translesion synthesis DNA polymerases [98].

Three unique amino acid insertions (insertions 1, 2 and 3) have been identified in both Pol1A and Pol1B that confer translesion activity. Two of these insertions exist in a domain that stabilizes exiting DNA and increases processivity of the polymerase (insertions 1 and 2) and the third resides in a domain that properly positions the template strand (insertion 3). These appear to be flexible elements as mutants lacking all three of these insertions are still able to synthesize DNA [98]. The translesion activity of Pol1A and Pol1B is negatively affected by the removal of insertions 1 and 3, indicating that these enzymes acquired translesion activity through evolution and the acquisition of these insertions.

### 4.3. DNA Unwinding

In *Arabidopsis,* a Twinkle DNA helicase/primase has been studied to determine its function and properties [72,99]. *Arabidopsis* also employs several gyrases to relieve tension in the replicating DNA molecules. There may be other DNA unwinding enzymes active in the organelles, but these have not been identified. Twinkle (T7 gp4-like protein with intramitochondrial nucleoid + localization) is similar to the bacteriophage T7 gp4 DNA primase/helicase protein and gets its name from Spelbrink et al, who noted that the protein fused to EGFP produced punctate fluorescence patterns similar to twinkling stars [100]. In *Arabidopsis* and T7 phage, Twinkle possesses a zinc finger domain that allows the protein to bind DNA and synthesize RNA primers for replication. In humans, amino acid changes to this area of the protein have removed the zinc finger domain and its priming ability [101]. However, in plants, both of these capabilities remain intact [67,72,99]. When compared to T7 gp4, *Arabidopsis* Twinkle has a slight extension between the primase and helicase domains when compared to phage gp4 and a longer N-terminal region. Twinkle localizes to both chloroplasts and mitochondria in plants.

*Arabidopsis* also possesses a truncated form of Twinkle referred to as Twinky (At1g30660). This truncation lacks the C-terminal helicase domain of Twinkle but maintains the primase domain. No work on Twinky has been published, and whether it is active in priming DNA or is simply a pseudogene is not confirmed.

One possible alternative DNA helicase in plants is DNA2 [102]. JHS1 in *Arabidopsis* is homologous to human and yeast nuclease/helicase DNA2. In humans and yeast, DNA2 cleaves at the junction between single stranded DNA (ssDNA) and double stranded DNA (dsDNA) at the base of a DNA flap [102]. Experiments with human DNA2 and DNA POLγ have shown a positive interaction and the ability to unwind DNA without cleaving the D-loop structure observed in human mitochondria [75]. In humans, DNA2 localizes to the nucleus and to mitochondria [74]. *Arabidopsis* DNA2 has not yet been shown to localize to either organelle. DNA2 is essential in humans, yeast, and *Arabidopsis* as mutations lead to a lethal phenotype [74,75,103], while its role in plants and plant mtDNA replication has yet to be defined [104].

### 4.4. Organelle DNA Gyrases

Research studying organellar gyrases is almost nonexistent; however, there is confirmation of gyrase A (GYRA) in *Arabidopsis* localizing to both chloroplasts and mitochondria. *Arabidopsis* also has two other gyrases that localize either to chloroplasts (GYRB1) or mitochondria (GYRB2) [89]. Disruption of any of these gyrase genes leads to a lethal phenotype. Depletion of DNA gyrase in *Nicotiana benthamiana* result in abnormal nucleoids, chloroplasts, and mitochondria. The role of the organellar gyrases in DNA replication is presently unknown [105]

### 4.5. Priming of DNA Synthesis

*Arabidopsis* may utilize Twinkle and RNA polymerase (RNAP) to prime organellar DNA replication. As mentioned previously, Twinkle is similar to T7 gp4 protein and possesses both DNA helicase and primase activities. Using Twinkle to prime organellar DNA synthesis is unique to plants, as animals utilize RNA polymerase to prime their mtDNA [106,107]. Nonetheless, plants do contain organellar RNA polymerases that could complement the activity of Twinkle [78,79].

Twinkle uses a unique recognition sequence to begin ribonucleotide synthesis and appears to prefer cytosine and guanine incorporation over uracil and adenine [99]. The recognition sequence is 5’-(G/C)GGA-3’ where the underlined nucleotides are cryptic. If either of the cryptic nucleotides or the guanine directly upstream from them are substituted, RNA synthesis is abolished. This is unique from other DNA primases, in that two cryptic nucleotides are required for synthesis whereas other primases often require one. The exact mechanism of Twinkle association with template DNA is not fully understood.

Twinkle preferentially incorporates CTP and GTP, which is curious as nearly all plant mitochondrial and chloroplast genomes are highly A/T rich [108]. Why then would a plant organellar primase preferentially incorporate CTP and GTP? One theory points to *Aquifex aeolicus*, a primitive thermophilic bacteria, in which primer synthesis is initiated from a trinucleotide sequence composed of cytosines and guanines much like *Arabidopsis* Twinkle [109]. This G–C rich sequence is hypothesized to provide stability during primer extension. Similarly, plants may rely on the stability of the template sequence 5’-(G/C)GGA-3’ paired with preferential CTP and GTP incorporation to provide thermodynamic stability. Another leading theory is that preferential incorporation of CTP and GTP aid in determining Okazaki fragment length [99].

The co-evolution of nuclear, plastid, and mitochondrial genomes in plants has led to an interesting arrangement of RNA polymerases (RNAP) in the organelles. Unlike animal mitochondria, which utilize a single RNA polymerase [110], plant organelles require multiple RNAPs: at least two for plastids and one for mitochondria. These genes are designated as *“RpoT”* genes, the *“T”* indicating their similarity to the single subunit RNA polymerases of T3 and T7 phage [111]. RNAP that targets to mitochondria are designated RpoTm and those that target to plastids are called RpoTp. RpoTmp represent RNAP that target to both organelles. Different species may possess multiple copies of these nuclear encoded organellar proteins, but the earliest phylogenetic versions of these enzymes exist in the waterlily *Nuphar advena*, a basal angiosperm [112]. All RNAPs in mitochondria are nuclear encoded, while plastids use both nuclear and plastid-encoded RNAPs. Extensive research has been performed on how plant RNA polymerases recognize promoters and transcribe genes. This portion of the review focuses on the potential RNAP has to prime DNA for synthesis rather than its role in transcribing genes.

Three single-subunit mitochondrial RpoT genes have been identified in *Arabidopsis*; however, only two have been proven to localize to mitochondria [79,111]. A duplication of one of these genes has led to the creation of a RpoTmp. How these enzymes coordinate synthesis of RNA is largely unexplored, although some research suggests RpoTmp is responsible for gene synthesis in early seedling development and RpoTm and RpoTp take over once the plant has fully developed [113]. 

In *Arabidopsis*, plastids require at least two RNAPs: nuclear encoded RNAP (NEP), and plastid encoded bacterial-like RNAP (PEP). The NEP and PEP versions of RNAP are distinct and do not share subunits [114]. The NEP is homologous to the phage-like single subunit RNA polymerases found in mitochondria. This RNAP is represented as RpoTp and is thought to be a duplication of the mitochondrial RpoTm. NEP isolated from *P. sativum* seems to act more like a primase than an RNAP [115], as it makes primers that are too short for transcription but much larger than other RNAPs. Like other RNAPs, it also showed preferential binding to single-stranded rather than double stranded DNA. The enzyme was also found to be resistant to inhibition by tagetitoxin, a specific inhibitor of chloroplast-encoded RNA polymerase, as well as polyclonal antibodies specific to purified pea chloroplast RNAP. These findings suggest that plastids and probably mitochondria possess an RNAP gene that functions as a DNA primase, although further research on this topic is needed.

Unlike the mitochondrial phage-like RNA polymerases, the PEP is made up of multiple subunits that share homology with the core subunits of *E. coli* RNAP: α, β, β’, and β’’. These subunits are encoded from the genes *rpoA*, *rpoB*, *rpoC1*, and *rpoC2*, respectively. In addition, several nuclear-encoded sigma factors form the PEP holoenzyme and provide promoter recognition for the plastid encoded subunits [76]. In agreement with the theory of endosymbiosis, the core enzyme of the NEP is also homologous to multi subunit RNA polymerases of cyanobacteria [111].

In humans and yeast, RNAPs are required to initiate and prime DNA for replication in mitochondria [106,107]. This makes sense when observing that Twinkle, a helicase-primase, is present in animals but lacks the primase activity observed in both phage and plants. Therefore, plants may also use their organellar RNA polymerases to prime DNA for synthesis. Unfortunately, the ability and scale on which this actually happens is grossly understudied, most likely due to the assumption that organellar DNA is primed by mimicking the simple replisome found in T7 phage. However, unlike animals in which mutation of Twinkle helicase/primase is embryo-lethal, plants with Twinkle knock-out mutations grow well, and display no phenotypic defects [116]. Therefore, the ability of RNA polymerase to prime DNA for synthesis may be extremely important to plants and could be a fruitful area of research.

### 4.6. Primer Removal

In *E. coli*, RNA primers are removed from DNA–RNA hybrids by the 5’-3’ exonuclease activity of DNA polymerase I. Since Pol1A and Pol1B lack 5’-3’ exonuclease activity, primer removal must be carried out by another enzyme. Recent work has shown RNase H-like activity both in mitochondria and chloroplasts [80]. In addition to RNase H, two exonucleases with homology to the 5’-3’ exonuclease domain of *E. coli* DNA polymerase I (5’-3’ EXO1 and 2) have also been predicted to localize to chloroplasts or mitochondria [68].

### 4.7. Single-Stranded DNA Binding Proteins

Plants utilize at least two types of single stranded DNA binding proteins (SSBs) in their organelles. The first one is similar to bacterial SSBs. *Arabidopsis* encodes at least two of these genes, called SSB1 and SSB2, although little is known about SSB2 [44,81]. SSB1 functions at replication forks by coating single stranded DNA to prevent fork collapse. This protein has been shown to localize to both mitochondria and chloroplasts, and stimulates bacterial RecA activity [39,44,81]. RecA is a bacterial protein involved in homologous recombination and strand invasion, and is discussed in greater detail below.

The second class of SSB proteins is called organellar single-stranded DNA binding proteins (OSB). OSB proteins are distinct from the bacterial SSB versions and are unique to plant organelles. At least four OSB genes are transcribed in *Arabidopsis* with OSB proteins localizing to both the mitochondria and chloroplasts. Although the function of these molecules has not been completely detailed, mutants for OSB1 accumulate mtDNA homologous recombination products. In subsequent generations, these products segregate into separate plant lines where one of the homologous recombination products is predominant. If OSB1 activity is restored the plants segregate into separate line they will revert to wild type configurations of mtDNA. However, if segregation has already occurred, restoration of OSB1 activity does not restore plants to wild type mtDNA configurations [82]. Therefore, OSB proteins are most likely involved in recombination surveillance and preventing transmission of incorrect recombination products to newly formed mitochondria.

In addition to OSB proteins, plants also utilize Whirly (WHY) and organellar DNA binding (ODB) proteins. WHY proteins form tetramers that take on the appearance of a whirligig, hence the name Whirly. There are three Whirly proteins—WHY1, WHY2, and WHY3 [83]. WHY1 and WHY3 localize to chloroplasts while WHY2 localizes to mitochondria. Whirly appears to have expanded roles from OSB proteins. Like OSB, WHY proteins also appear to be involved in recombination surveillance and have also been shown to associate with RNA molecules. Some WHY proteins have been associated with double stranded DNA repair [84,85] and regulation of defense genes in response to pathogens [117].

### 4.8. DNA Recombination

As mentioned in the previous section, OSB, ODB, and WHY proteins have been shown to participate in DNA repair in mitochondria and chloroplasts. In addition, plant organelles also contain proteins involved in homologous DNA recombination. There are two classes of proteins dedicated to recombination in plant organelles. One is a MutS homolog called MSH1, and the others are RecA homologs.

MutS from *E. coli* is part of the mismatch repair (MMR) pathway and corrects point mutations and small insertions and deletions by preventing recombination between partially homologous DNA sequences. MSH1 is a nuclear encoded gene and mutants display patchy green/white/yellow leaves symptomatic of dysfunctional chloroplasts, with a non-Mendelian inheritance pattern [118]. Due to this activity, this gene was originally called *chm* for *chloroplast mutator*. Later, it was discovered that *chm* mutants cause rearrangements to the mitochondrial genome that lead to the observed phenotypes and defective chloroplasts. Despite extensive searching, no mutation or rearrangement of the plastid genome has been observed in *chm* mutants [119]. Being homologous to MutS from *E. coli*, the gene was consequently renamed MSH1 and mutants designated as *msh1* plants [120].

Insertion mutations of yeast *msh1* lead to a petite phenotype indicative of mitochondrial dysfunction. Mutation of yeast *msh1* is also accompanied by large-scale point mutations and rearrangements in the mitochondrial genome [121]. Interestingly, plant MSH1 mutants do not accumulate point mutations over time, suggesting that the plant MSH1 specializes primarily in recombination and is not essential for correcting mismatches.

*E. coli* utilizes the adaptor protein MutL and endonuclease MutH to assist in the mismatch repair pathway, but no homologs of these proteins have been identified yet in plant organelles. Instead, plant MSH1 possesses three recognizable domains and three unknown domains to facilitate mismatch repair. These include a conserved DNA binding and mismatch recognition domain and an ATPase domain homologous to those in *E. coli* MutS. Point mutations to the ATPase and endonuclease domains of plant MSH1 led to the defective chloroplast phenotype [122], suggesting that plant MSH1 may provide mismatch recognition and base excision without the need for MutL or MutH homologs, although this has not been experimentally shown. Recent studies have shown that MSH1 suppresses homeologous recombination [123,124]. Plant MSH1 also has a unique GIY-YIG endonuclease domain, which binds specifically to the D-loop structure, suggesting that this protein may recognize and resolve mismatch-containing intermediates [125].

RecA facilitates homologous recombination by correctly pairing homologous sequences and promoting strand invasion. Eukaryote versions of this protein are called RAD51. All homologous recombination begins with strand invasion mediated by RecA family proteins, making this protein crucial for this type of repair. RecA functions by coating single stranded DNA at lesions to form presynaptic filaments. This complex will then search for homology within intact double stranded DNA. Once homology has been established, the presynaptic complex will destabilize the double stranded DNA promoting strand exchange and D-loop formation. Three RecA proteins have been identified in *Arabidopsis*: RecA1, RecA2, and RecA3. RecA1 localizes to plastids, RecA3 to mitochondria, and RecA2 to both organelles [86]. Mutations to RecA3 cause mitochondrial rearrangements distinct from those observed in MSH1 mutants. The rearrangements observed in RecA3 mutants are due to homologous recombination of repeated sequences in the mitochondrial genome. Reintroducing RecA3 into these mutants results in a reversal of this effect in most of the progeny by abolishing aberrant mitochondrial DNA molecules. RecA1 and RecA2 appear to be even more essential to homologous recombination as mutations in these genes cause a seed-lethal phenotype. This may be explained by the lack of the C-terminal domain found in both RecA1 and RecA2 as well as bacterial homologs. In bacteria, deletion of this C-terminal domain enhances the activity of RecA, suggesting its involvement in autoregulation.

### 4.9. DNA Ligation

The amount of information surrounding plant organellar DNA ligases is extremely limited. Unlike mitochondria, no DNA ligase has been confirmed or observed functioning in plastids, representing a potential avenue of research, as the activity of this enzyme in both organelles must be present. Four DNA ligase genes have been identified in the genome of *Arabidopsis*; however, only DNA ligase 1 (LIG1) has been identified in mitochondria [126]. Plant LIG1 knockouts are seedling-lethal and knockdown mutants display severe growth defects due to effects on the nuclear genome rather than the mitochondrial genome [127].

## 5. Conclusions

Genomes found in plant mitochondria and chloroplasts are essential for organelle function, but there is still relatively little known about how these genomes are replicated and maintained. This is especially true for plant mitochondria, which have a very large variation in genome size depending on the species, and there is considerable evidence that the genome exists as linear subgenomic molecules, raising questions as to how the integrity of the genetic information is maintained. Thus, plant mitochondrial genomes and their replication are much more complex than their animal counterparts. It is clear that for at least some replication functions more than one gene is present in *Arabidopsis*, suggesting the possibility of functional redundancy. For chloroplasts, although a DNA replication mechanism has been established, it is quite possible that more than one mechanism is involved, perhaps for different stages of growth or in response to different signals. Further research is needed to better understand the basic structure of the organelle genomes and how these DNA molecules are replicated. In addition, the mechanism(s) for maintaining genome copy number and regulation of replication initiation are not known and should be studied.

## Figures and Tables

**Table 1:** Proteins involved in plant organellar DNA replication.

Function	Protein Name	TAIR	Homology	Localization *	Ref.
DNA polymerase	Pol1A or Pol gamma 2	At1g50840	Bacterial	M, P	[69,70,71]
	Pol1B or Pol gamma 2	At3g20540	Bacterial	M, P	[69,70,71]
Helicase	Twinkle	At1g30680	Phage	M, P	[72,73]
	DNA2	At1g08840	Mammalian	?	[74,75]
Priming	Twinkle	At1g30680	Phage	M, P	[72,73]
	RNA polymerase				[76,77,78,79]
	RpoT1	At1g68990	Phage	M	
	RpoT2	At5g15700	Phage	M, P	
	RpoT3	At2g24120	Phage	P	
	RpoA	AtCg00740	Bacterial	P	
	RpoB	AtCg00190	Bacterial	P	
	RpoC1	AtCg00180	Bacterial	P	
	RpoC2	AtCg00170	Bacterial	P	
Primer Removal	RNaseH				
	AtRNH1B	At5g51080			[80]
	AtRNH1C	At1g24090	Bacterial	M, P	[80]
	EXO1	?	Bacterial	P	[68]
	EXO2	?			[68]
ssDNA binding, recombination monitoring	SSB1	At4g11060	Bacterial	M, P	[44,81]
	SSB2	At3g18580	Bacterial	M	[44]
	OSB1	At3g18580	Bacterial-like, but unique to plants	M	[82]
	OSB2	At4g20010	Unique to plants	P	[44]
	OSB3	At5g44785	Unique to plants	M, P	[77,82]
	OSB4	At1g31010	Unique to plants	M	[82]
	WHY1	At1g14410	Unique to plants	P	[83,84,85]
	WHY2	At1g71260	Unique to plants	M	[83,84,85]
	WHY3	At2g02740	Unique to plants	P	[83,84,85]
	ODB1	At1g71310		M, N?	
	ODB2	At5g47870		P, N?	
Recombination	RecA1	At1g79050	Bacterial	P	[86]
	RecA2	At2g19490	Bacterial	M, P	[86]
	RecA3	At3g10140	Bacterial	M	[86,87]
	MSH1	At3g24320	Bacterial	M, P	[88]
Topoisomerase	Topoisomerase I	At4g31210	Bacterial	M, P	[77]
	DNA Gyrase A	At3g10690	Bacterial	M, P	[89]
	DNA Gyrase B1	At3g10270	Bacterial	P	[89]
	DNA Gyrase B2	At5g04130	Bacterial	M	[89]
	DNA Gyrase B3	At5g04110	Eukaryotic	N	[89]
Ligation	LIG1	At1g08130	Bacterial	M, N	[44]

* Localization to M (mitochondria), P (plastids), or N (nucleus). ? = unknown or potential localization.
